# Functional Role of P-Glycoprotein and Binding Protein Effect on the Placental Transfer of Lopinavir/Ritonavir in the Ex Vivo Human Perfusion Model

**DOI:** 10.1155/2009/726593

**Published:** 2009-05-18

**Authors:** Pierre-Francois Ceccaldi, Laurent Gavard, Laurent Mandelbrot, Elisabeth Rey, Robert Farinotti, Jean-Marc Treluyer, Sophie Gil

**Affiliations:** ^1^Department of Obstetrics and Gynecology, Louis Mourier Hospital, AP-HP, Paris 7 University, 92701 Colombes, France; ^2^Faculty of Pharmacy, University Paris-Sud, EA2706, 92296 Châtenay-Malabry, France; ^3^Department of Pediatric Pharmacology, Saint Vincent de Paul Hospital, AP-HP, Paris 5 University, 75014 Paris, France; ^4^Department of Pharmacy, Pitié-Salpétrière Hospital, AP-HP, 75013 Paris, France

## Abstract

*Aims*. To study the influence of P-glycoprotein (P-glycoprotein, ABCB1, MDR1) function on placental transfer of lopinavir with ritonavir at different albumin concentrations. *Methods*. Cotyledons were perfused with lopinavir, ritonavir, and the internal control antipyrin, at various albumin concentrations (10, 30, 40 g/L). After the control phase of each experiment, the P-glycoprotein inhibitor ciclosporin A was added at middle perfusion (45 minutes). Fetal Transfer Rate (FTR) and Clearance Index (CLI) were compared between the 2 phases. *Results*. In the control phase, the clearance index of lopinavir decreased from 0.401 ± 0.058 to 0.007 ± 0.027, as albumin concentrations increased from 10 g/L to higher concentrations (30, 40 g/L). When adding ciclosporin A at physiological albumin concentrations, the clearance index of lopinavir increased significantly 10.3 fold (95% of CI difference [−0.156, −0.002], *P* = .046) and became positive for ritonavir. *Conclusions*. Even at high albumin concentrations, inhibition of placental P-glycoprotein increased placental transfer of lopinavir, suggesting that this efflux pump actively reduces placental transfer of the drug. This mechanism may play a role in fetal exposure to maternal antiretroviral therapy.

## 1. Introduction

**What is Already Known about this Subject?**

We founded previously that albumin concentration had a strong impact on placental transfer of lopinavir and ritonavir. When the concentration of albumin was increased to 40 g/L, we observed a marked decrease of placental transfer for both ritonavir and lopinavir.

Our findings suggest that P-glycoprotein in placentas from HIV-infected women would contribute to modulate the maternofetal transport of antiretrovirals across the placental barrier and consequently diminish fetal exposure to these compounds.

**What this Study Adds?**

This study uses the well-known human cotyledon-perfused model to investigate the P-glycoprotein effect on the placental transfer of lopinavir and ritonavir at low (10 g/L) and physiologic (30 and 40 g/L) albumin concentrations. Each placenta is its own control and ciclosporin A was coperfused at middle time of the perfusion. Expression on placenta of P-glycoprotein was analyzed.

The clearance index of lopinavir and ritonavir increased following the addition of ciclosporin, even at physiologic albumin concentration. Expression on placenta of P-glycoprotein was wide, and there was no significative correlation with clearance index of lopinavir.

Lopinavir coformulated with low-dose ritonavir (Kaletra ®, Abbott) is commonly used in first-line combination antiretroviral therapies for HIV-infected patients. It is also recommended for the prevention of mother-to-child transmission because of its capacity to reduce HIV-1 RNA concentrations to below the detection limit in a few weeks [1]. However, in view of fetal toxicity observed in animal studies, lopinavir/ritonavir is classified under category C by the US Food and Drug Administration (FDA). The degree of concern about its safety to the fetus depends on the amount of placental transfer of the drug. Using the ex vivo perfused human placental cotyledon, we reported for lopinavir a placental transfer rate of 3.3% ± 0.5 in the presence of physiological albumin concentrations [2]. In a clinical study of paired cord and maternal blood at delivery, the ratio was 0.2 ± 0.13 [3]. These concordant experimental and clinical data suggest that the fetus is usually exposed to relatively low concentrations of lopinavir during maternal therapy.

The placental transfer of drugs with molecular weights 800 Da such as lopinavir and most antiretovirals is known to be influenced by various physicochemical properties and by the amount of protein binding. There is now a large body of evidence suggesting that efflux proteins play an important role in reducing the amount of placental transfer of some drugs. P-glycoprotein (P-glycoprotein, ABCB1) is an active ATP-dependent drug-efflux pump highly expressed in the maternal facing cell membrane of trophoblasts [4]. This protein seems to play an important role in limiting the maternal-fetal transfer of protease inhibitors (PIs). Recently a study has shown a 3.3-fold increased expression of P-glycoprotein in placentas of HIV-infected women. It could be theoretically associated with a decreased transfer of substrates of P-glycoprotein, such as PI [5]. Another study reported a higher expression of P-glycoprotein in early of pregnancy than at term, which may have an impact on placental transfer of PI-like lopinavir, which is a P-glycoprotein substrate [6]. A study on cultured placental choriocarcinoma cells highlighted that P-glycoprotein inhibitor ciclosporin A could increase the cellular uptake of the P-glycoprotein substrates [7]. Placental P-glycoprotein seems to play a protective role for the fetus against drugs and xenobiotics [8].

The goal of this work was to study the influence of the activity of P-glycoprotein and the concentration of perfused albumin on the transplacental passage of lopinavir and ritonavir on the experimental model of the perfused human cotyledon.

## 2. Methods

### 2.1. Materials

Placentas were perfused in an open double circuit according to a method modified from that of Schneider and Huch as previously described [9, 10]. Placentas were collected after uneventful term (37–42 weeks gestational age) vaginal deliveries from HIV-seronegative women receiving no drug treatment, except oxytocin or epidural anesthesia during labor. Each patient gave written informed consent to participate in the study. The maternal and fetal solutions were prepared with Earle medium with 10, 30, or 40 g/L bovine serum albumin (Euromedex, France). Lopinavir and ritonavir were used in a powder base provided by Abbott Laboratories (Rungis, France). The targeted concentrations infused in the maternal compartment were 6 mg/L for lopinavir and 3 mg/L for ritonavir. Ciclosporin A (10 *μ*M) is a well-studied molecule which has an inhibitory effect on P-glycoprotein and may be used with caution during pregnancy [11].

### 2.2. Placental Perfusion Technique

Perfusion experiments were started within 20 minutes after delivery. After a visual examination for lack of evident lesions on the chorionic plate, a truncal branch of the chorionic artery supplying a selected cotyledon and the associated vein was cannulated. The fetal circulation was established at a flow rate of 6 mL/min in order to ensure a balance between arterial and fetal venous flows. Placentas with evidence of vascular leakage were discarded. On the maternal side the perfused area progressively whitened, which allowed visualization of the chosen cotyledon. The cotyledon was placed in the perfusion chamber with the maternal surface upward. The perfusion chamber was maintained at 37°C through a circulating thermostat bath. The intervillous space on the maternal side was perfused by two needles piercing the basal plate. The maternal flow rate was 12 mL/min. The pH was adjusted to 7.4 ± 0.1 for the maternal and 7.3 ± 0.1 for the fetal solutions, respectively, by the addition of a solution of sodium bicarbonate and hydrochloric acid. The perfusion length was 95 minutes. Combination of lopinavir, ritonavir, and a freely diffusing marker antipyrin (20 mg/L) (Sigma, France), were perfused into the maternal reservoir. Ciclosporin A was added at middle time of the perfusion, that is, 45 minutes. Samples from the maternal and fetal solutions were collected in order to determine concentrations of antipyrin, lopinavir, and ritonavir at 25, 30, 35, 40 minutes for the control phase and 75, 80, 85, 90 minutes for the ciclosporin A phase.

### 2.3. Drug Analysis

Lopinavir and ritonavir were measured simultaneously on a 100 *μ*L sample to which an internal standard (clazepam) was added. The extraction was performed with ethyl acetate in alkaline medium. The range of concentrations was 0.2 to 20 mg/L for lopinavir and 0.05 to 5 mg/L for ritonavir. The drugs were separated isocratically on a nucleoside C8 3 *μ*m (F 67722; 125∗4.6 mm; Macherey-Nagel Hoerdt, Hoerdt, France) with buffer (tetramethylammonium perchlorate 0.01 mol/L in a 0.01% trifluoroacetic acid (pH = 3) and acetonitrile [60 : 40]). Ultraviolet absorbance at 205 nm was used for detection. The lower limits of quantification (LOQ) were 0.2 and 0.05 for lopinavir and ritonavir, respectively. Mean intra-assay precision at the low-quality control was lower than 12.5% and accuracy (percent deviation from expected) at the LOQ was less than 10%. The overall recovery was 80%.

### 2.4. Pharmacokinetic Analysis

The results were reported as a Fetal Transfer Rate (FTR = Cf/Cm, Cf as fetal concentration and Cm as maternal concentration) and as a Clearance Index (ClI = FTRcompound/FTRantipyrin) [12].

### 2.5. P-glycoprotein Expression on Placentas

This analysis was previously used to study the quantitative expression during pregnancy [6]. Trophoblast cells were scrapped, centrifuged, and incubated in a lysis buffer containing 10 mM Tris-HCl (pH 7.4), 5 mM EDTA (pH 8), 126 mM NaCl, 1% triton, 0.1% SDS, protease inhibitors (1 mM PMSF, 5 *μ*g*·*mL^−1^ pepstatin, 0.5 mg*·*mL^−1^ leupeptin, 31.2 mg*·*mL^−1^ benzamidine, 5 *μ*g*·*mL^−1^ aprotinin) for 30 minutes at 4°C. After incubation, the suspension was centrifuged at 12 000 g for 20 minutes at 4°C. Protein concentration was determined using the colorimetric Bicinchoninic Assay kit (Sigma-Aldrich, Saint-Quentin Fallavier, France), with bovine serum albumin as standard. Ten micrograms of proteins were subjected to SDS-polyacrylamide gel electrophoresis with an 8% polyacrylamide gel and transferred onto a nitrocellulose membrane (Amersham, Orsay, France). The nitrocellulose membranes were then incubated with primary antibody (C219, diluted 1 : 100; DAKO, Denmark) followed by a peroxidase-conjugated antimouse IgG secondary antibody (diluted 1 : 7500; DAKO, Denmark). The immunoreactive bands were visualized by the Enhanced Chemo-Luminescent system (Perkin Elmer Life Science, Boston, MA, USA). The amount of immunoreactive P-glycoprotein on placental samples was relatively expressed to the signal measured in K562R7 extracts. The autoradiographs of P-glycoprotein protein were scanned and analyzed by densitometry using the Scion Image program to obtain a quantitative evaluation of the levels in the placenta.

### 2.6. Statistical Analysis

A paired Student's *t*-test test was performed to compare datas, expressed as means ± SD, for the clearance index comparing control and ciclosporin A phases. An unpaired *t*-test was performed to compare as different albumin concentrations. The Spearman test to correlate the clearance index and placental expression of P-glycoprotein was used. An *α* risk value of less or equal to 0.05 was considered as significant. Statistical analyses were performed using Graphpad Prism 4 for Windows version (Graphpad Software, USA).

## 3. Results

The open-open system was used to determine the transfer rates of lopinavir and ritonavir under steady-state conditions. Eleven placentas were validated for this perfusion. The maternal concentrations were, respectively, 6.121 ± 1.824 mg/L for lopinavir and 3.318 ± 1.134 mg/L for ritonavir. The variation of fetal transfer rate of antipyrin in the control and ciclosporin A phases (mean of values at 25, 30, 35, and 40 minutes versus 75, 80, 85, and 90 minutes) was collected at steady state. Mean fetal transfer rates of antipyrin (31.7% ± 6.31) with ciclosporin A and without (25.3% ± 4.51) at 10 g/L (95% CI of the difference [−1.67, 14.5], *P* = .076) and 30 and 40 g/L (28% ± 6.91 versus 25.4% ± 8.48, 95% CI of the difference [−4.28, 9.59], *P* = .396) were not significantly different.

### 3.1. Albumin Effect

**Figure 1:**
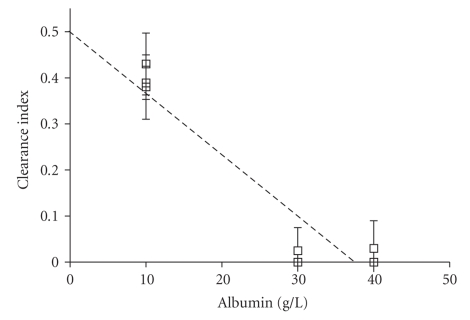
Variation of clearance index of lopinavir at different albumin perfused concentrations (for 10 g/L *n* = 3, for 30 g/L *n* = 3 and for 40 g/L *n* = 5). Linear regression (*r* ^2^ = 0.886).

Five placentas were perfused with 40 g/L bovine serum albumin concentration, 3 with 30 g/L, and 3 with 10 g/L (Table 1, Figure 1). The clearance index for lopinavir was significantly lower in 30 or 40 g/L albumin concentrations than 10 g/L, (10 versus 40 g/L, 95% CI [0.360, 0.427], *P* < .0001; 10 versus 30 g/L, 95% CI [0.343, 0.440], *P* < .0001). At higher albumin concentrations there was no difference (30 versus 40 g/L, 95% CI [−0.022, 0.027], *P* = .822). Clearance index of ritonavir was low at 10 g/L of albumin (0.060 ± 0.089) and was zero at 30 or 40 g/L albumin concentrations.

### 3.2. Placental Transfer of the Lopinavir/Ritonavir Combination with Ciclosporin A (Figure 2)

At 10 g/L albumin concentration, the clearance index of lopinavir increased significantly following the addition of ciclosporin A (95% CI [−0.197, −0.048], *P* = .02). At physiological concentrations of albumin (30 and 40 g/L), the difference was significant (95% CI [−0.156, −0.002], *P* = .046). The clearance index of ritonavir increased in the presence of ciclosporin A but it was not significant.

### 3.3. Effect of Placental P-Glycoprotein (Table 2, Figure 3)

Expression on placenta of P-glycoprotein was wide (3.06 ± 2.384, covar = 77.91%). There was a low and no significant correlation between expression of the P-glycoprotein and clearance index of lopinavir without ciclosporin A (*r* = 0.109).

## 4. Discussion

In this study, pharmacologic blockade of P-glycoprotein by ciclosporin A significantly increased the placental transfer of lopinavir. This finding strongly suggests that under physiological conditions, P-glycoprotein plays an active role in decreasing placental transfer of lopinavir from the mother to the fetus. This is consistent with studies in the rat, which concluded that P-glycoprotein produced an active and functional barrier between the maternal and fetal circulations [13].

Our findings are also consistent with those of Molsa et al. on the placental transfer of saquinavir [14]. In this study, preperfusion with PSC833 or GG918, two inhibitors of the activity of P-glycoprotein, increased the placental transfer of saquinavir by 7.9-fold and 6.2-fold, respectively, compared with control placentas. However, PSC833 had no effect on the transfer of saquinavir from the fetal to the maternal direction. In our case, controls were placentas themselves like recent study realized by Sudhakaran, which eliminates biases such as differences between placentas for various genetic and physiologic reasons [15]. Recently, Agarwal et al. confirmed on specific cell cultures that both P-glycoprotein and MRP2 mediate transport of lopinavir [16]. MRP2 has low expression on the human placenta and Sudhakaran et al. suggested for this transporter was unlikely to contribute significantly to limiting the transfer of these drugs to the fetus [15].

Previously, we showed that the mean expression of P-glycoprotein in human placentas progressively decreases by twofold decrease between the first and third trimesters [6]. Also, many environmental factors could affect the P-glycoprotein expression [17]. HIV infection itself may play a role as suggested in a study by our group who compared 35 full-term placentas from uninfected women and 24 from HIV-1 infected women and showed a significant 3.3-fold mean increase in MDR1 placental transcription in HIV-infected women. In this research, there did not appear to be intraplacental variations of expression of P-glycoprotein [5]. Our study lacked power to conclude for an high correlation between variation of expression of placental P-glycoprotein with its activity. Relation to genetic polymorphism of the MDR-1 gene may play role [18]. The reported effects of MDR1 SNPs have been inconsistent and, in some cases, conflicting [19]. This may be due to a number of potential confounding factors of the observed MDR1 polymorphism effect in vivo issues, as suggested by Marzolini et al. [19], including MDR1 haplotypes, environmental factors, and study design.

In our study, the albumin concentration in the perfusate stands out as an important factor in transplacental transfer of lopinavir. This confirms previous results from our group [2]. At low albumin concentrations, the transfer of lopinavir and ritonavir was compatible with passive diffusion, whereas the clearance decreased significantly at physiological albumin concentrations (30 and 40 g/L). Sudhakaran et al. determined the protein binding characteristics of two PIs, indinavir and saquinavir [20, 21]. They were examined in maternal and umbilical cord plasma. Both PIs were significantly less bound in umbilical cord than maternal plasma, indicating that the low cord-mother ratios of total drug were influenced by differential protein binding and the nature of this binding protein (alpha1-acid glycoprotein, human serum albumin).

Our findings indicate that P-glycoprotein plays a role in the human placental barrier, even at physiological albumin concentration. This may have important clinical implications. In particular interaction with P-glycoprotein may lead to difference in fetal exposure to maternally administrated drugs. For instance, calcium blockade currently used for tocolysis or hypertension during pregnancy is P-glycoprotein inhibitor, which may increase the transfer of P-glycoprotein substrates by several folds [22–24].

## Figures and Tables

**Figure 2:**
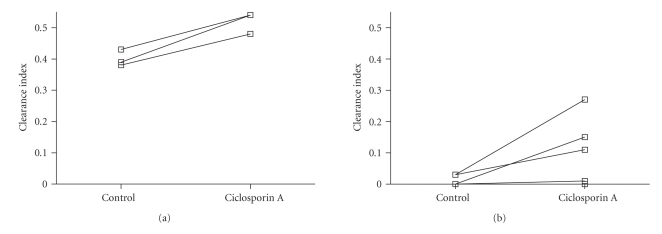
Variation of clearance index of lopinavir in the control and ciclosporin A phases (mean of values at 25, 30, 35, and 40 minutes versus 75, 80, 85, and 90 minutes) at low ((a), 10 g/L) and physiologic ((b), 30, and 40 g/L) albumin concentrations. The lines join the data from the same placenta (*n* = 3 at 10 g/L, *n* = 3 at 30 g/L and *n* = 5 at 40 g/L). Mean clearance index of lopinavir (0.401 ± 0.058) with ciclosporin A (0.521 ± 0.066) at 10 g/L (95% CI of the difference [−0.186, −0.054], *P* = .016) and 30 and 40 g/L (0.007 ± 0.027 versus 0.072 ± 0.101, 95% CI of the difference [−0.156, −0.002], *P* = .046).

**Figure 3:**
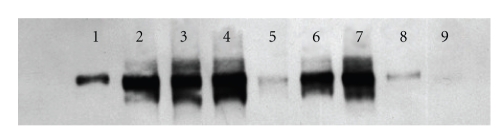
Western blot of the P-glycoprotein on placenta perfused at 30 and 40 g/L of albumin (*n* = 8). Control was on the left. Sample 1 was control sample; sample 9 was not visible at this time of exposition.

**(a):** 

**A**	Control	Ciclosporin A	Relative increase in ClI
Albumin 10 g/L	0.401 ± 0.058	0.521 ± 0.065	×1.3*
Albumin 30 and 40 g/L	0.007 ± 0.027	0.072 ± 0.101	×10.29*

**(b):** 

**B**	Control	Ciclosporin A	Relative increase in ClI
Albumin 10 g/L	0.06 ± 0.104	0.104 ± 0.181	×1.73
Albumin 30 and 40 g/L	0	0.071 ± 0.103	—

**Table 2:** Clearance index of lopinavir in the control phase (0–45 minutes) and Pgp placental expression for experiments during the control phase at physiological albumin concentrations (30–40 g/L).

Sample	1	2	3	4	5	6	7	8	9
Relative Pgp expression	1	3.1	3.14	3.59	0.68	2.71	3.38	0.74	0.31
ClI lopinavir	—	0	0	0.03	0	0	0	0	0.025

Pgp: P-glycoprotein, ClI: clearance index. Sample 1: control cell line R7. Samples 2–9: relative expression to sample 1 expression.
